# The practices of Tunisian psychiatric residents in sexology: A cross-sectional study

**DOI:** 10.1192/j.eurpsy.2026.12107

**Published:** 2026-07-31

**Authors:** S. Walha, S. Hamzaoui, R. Hosni, A. Ouertani, A. Aissa, R. Jomli

**Affiliations:** Psychiatric department “A”, RAZI Hospital, Mannouba, Tunisia

## Abstract

**Introduction:**

Sexuality is a fundamental human need. Contrary to popular belief, sexual activity is maintained in patients with mental illness, whose sexual interest and desire evolve with treatment without disappearing. Despite the importance of sexual health for patients receiving psychiatric care, communication about sexuality between doctors and patients faces several obstacles and shortcomings. Among these limiting factors are the practices of psychiatric residents with regard to patient sexuality.

**Objectives:**

The objectives of our study were to evaluate the practices of psychiatric residents with regard to patient sexuality in Tunisia and to determine the factors associated with the nature of these practices in this population.

**Methods:**

This was a descriptive and analytical study with cross-sectional data collection conducted on a population composed of a sample of psychiatry residents practicing in Tunisia at all levels: from the first to the fifth year. Our study was based on an online questionnaire and assessed participants’ Practices of sexuality. It took place from March 1 to April 1, 2025.

**Results:**

Forty-two young psychiatrists were identified. The average age of these young practitioners was 27.95 years. The majority of psychiatrists participating in the study were female (83%; n=35). The majority of participants (31%) were fourth-year psychiatry residents. The pharmacological approaches that psychiatrists often used to treat SDs were mainly the prescription of IPDE5 (often or sometimes prescribed by 26% and 12% of practitioners, respectively). Specific sexological approaches were only practiced by a minority of psychiatrists (only 17% for CBT and 33% for couples therapy), with the exception of supportive psychotherapy and marital counseling (43% of psychiatrists) and sexual information provided by 71% of psychiatrists. Regarding the factors influencing doctors’ practices, we found a significant negative relationship between gender and pharmacological approaches (p=0.001). We observed a statistically significant difference between the different stages of residency in terms of pharmacological approaches to patients’ sexuality (p=0.001). Furthermore, a significant relationship was found between pharmacological approaches and residents with degrees in sexology (p=0.01). Finally, a significant relationship was found between pharmacological approaches and patient satisfaction (p=0.023).

**Conclusions:**

There are several gaps in Tunisian doctors’ practices regarding their patients’ sexuality. At the end of this study, we suggest the generalization of sexology education in all medical schools in Tunisia and, above all, the participation of residents in specific sexology consultations. This will promote the good sexual health of our patients and contribute to better mental balance.

**Disclosure of Interest:**

None Declared

